# New approaches in human health risk assessment

**DOI:** 10.3402/ijch.v75.33845

**Published:** 2016-12-13

**Authors:** Khaled Abass, Anders Carlsen, Arja Rautio

**Affiliations:** 1 Centre for Arctic Medicine, Thule Institute, University of Oulu, Oulu, Finland; 2 Research Unit of Biomedicine, University of Oulu, Oulu, Finland; 3 Department of Pesticides, Menoufia University, Egypt; 4 Department of Public Health, Aarhus University, Aarhus, Denmark

**Keywords:** environmental pollutants, ArcRisk, exposure limit, Toxicokinetic modelling, *In vitro* mechanistic studies

## Abstract

Studies on the precise impact of environmental pollutants on human health are difficult to undertake and interpret, because many genetic and environmental factors influence health at the same time and to varying degrees. Our chapter in the AMAP report was based on new approaches to describe risks and future needs. In this paper, we will introduce the issues associated with risk assessment of single chemicals, and present suggestions for future studies as well as a summary of lessons learned during the health-related parts of the European Union-funded FP7 project ArcRisk (Arctic Health Risks: Impacts on health in the Arctic and Europe owing to climate-induced changes in contaminant cycling, 2009–2014; www.arcrisk.eu).

Humans are exposed to toxic substances via ingestion, inhalation and dermal absorption. The risk assessment process is generally used for evaluating non-cancer hazard and cancer risk in environmental and occupational settings. This methodology considers each external source of a contaminant uniquely with its own characteristics (1). On the contrary, the concentration of the contaminant in blood generally provides the sum of exposure from various routes. Several reference values have been published by different organizations to evaluate the exposure limit (Table I). There may be synergistic effects when more than one pollutant is present at the same time (14). For a few substances, a direct association has been documented between a disease and exposure to a chemical; this is typically the case for chemicals used for a long time and are now banned or in the process of being banned.

**Table I:** Biological guideline values for contaminants in blood

Contaminants	Media	Comments	Guideline values	Reference

Total PCBs	Plasma lipids	- For pregnant women, women of childbearing age, breastfeeding women	0.7 µg/g	(2)
		- Young girls and teenage girls		
		- Children under age 3		
		- Boys >3 years, adults beyond childbearing age	1.8 µg/g	
Pb	Blood	- Canadian blood-lead intervention level	100 µg/l	(3)
		- Pregnant women intervention level	50 µg/l	(4)
		- Children (reference level)	50 µg/l	(5)
MeHg	Blood	- Reference dose	5.8 µg/l	(6)
		- Intervention level: children, pregnant women and women of childbearing age	8 µg/l	(7)
		- Females (≥50 years) and males (>18 years) at increased risk	≥20 µg/l	(8)
		- Females (≥50 years) and males (>18 years) at risk	≥100 µg/l	(8)

Reference values for cadmium and mercury in blood or urine (9,10). Reference values are statistically derived values that indicate the upper margin of background exposure to a given pollutant in a given population at a given time. Reference values are derived from the analytical data provided within the framework of the German Environmental Surveys (11).				

Contaminants	Media	Comments	Reference values (µg/l)	

Cd	Urine	Children (6–12 years)	0.5	
		Non-smoking adults (18–69 years)	0.8	
Cd	Blood	Children (6–12 years)	0.5	
		Non-smoking adults (18–69 years)	1.0	
Hg	Urine	Children (6–12 years) without amalgam filling	0.7	
		Adults (18–69 years) without amalgam filling	1.0	
Hg	Blood	Children (6–12 years), fish consumption ≤3 times/month	1.5	
		Adults (18–69 years), fish consumption ≤3 times/month	2.0	

Human biomonitoring values (HBVs) for cadmium and mercury in blood and urine (12,13). HBM are derived on the basis of toxicological and epidemiological studies. HBM I represents the concentration of a substance in humans below which there is no risk or adverse health effects and no need for action. HBM II represents the concentration of a substance in humans above which there is an increased risk for adverse health effects and urgent need to reduce the exposure and to provide individual biomedical care (advice). This is considered as an intervention or action level.				

Contaminant	Media	Comments	HBM I	HBM II

Cd	Urine	Adults ≤25 years	1.0 µg/g creatinine	3.0 µg/g creatinine
		Adults >25 years	2.0 µg/g creatinine	5.0 µg/g creatinine
Hg	Blood	Children and adults	5 µg/l	15 µg/l
Hg	Urine	Children and adults	5 µg/g creatinine, 7 µg/l	20 µg/g creatinine, 25 µg/l

## Exposure of mercury and persistent organic pollutants in the ArcRisk cohorts

One of the tasks in the ArcRisk project was to compare Hg levels measured in Arctic cohorts with those from the Mediterranean region (belongs to the mercury belt area) where populations are exposed to low levels of Hg throughout their lifespan. The levels of Hg in the Arctic populations have decreased to the same levels as found in Italy, Slovenia, Croatia, Arctic Norway, Spanish Islands and Greece (mean Hg levels<100 µg/l), but there was high variation in measured Hg concentrations between individuals in all cohorts. Humans in Europe and in the Arctic are exposed to Hg mainly through fish consumption. Other sources such as elemental Hg in air and inorganic Hg in food items are minor sources of exposure (15).

Among the ArcRisk cohorts, those living in the coastal region in Norway, Greece and Slovenia had higher levels than those living in the inland or central European regions (16,17). The source of fish (imported, farmed or local) may have an impact in the Mediterranean region, since those consuming local fish had higher Hg levels. Mercury levels in fish vary greatly according to species and origin (16,18). Farmed fish generally contain less Hg than free-ranging fish from the open ocean.

Neurodevelopment among children in the Mediterranean coastal regions of Italy, Slovenia, Croatia and Greece was investigated in the PHIME project, and it was found that the Hg levels measured were low and did not significantly affect neurodevelopment by the age of 18 months (19). Instead, higher fish consumption during pregnancy was associated with higher cognitive and language (but not motor) neurodevelopmental performance at that age (15,16).

Blood levels of persistent organic pollutants (POPs) analysed declined in all other ArcRisk cohorts except in Russian indigenous peoples; there were no changes in geometric mean serum concentration of polychlorinated biphenyls (PCBs) in children and adults over the 10-year period. Concentration levels and enantiomeric fractions for α-hexachlorocyclohexane (α-HCH) and *trans*-, *cis*- and oxychlordane in selected Greenlandic traditional food items collected at the local market in Nuuk in 2010 were below the tolerable daily intake (TDI) threshold (20). Furthermore, daily exposure to PCBs, polybrominated diphenylethers and perfluorinated alkylated substances from traditional Greenlandic seafood items was below the TDI for all compounds. Excluding local traditional food items such as intestines and blubber from the diet has a strong positive effect on reduction of POP levels.

The median levels of PCB153 in serum were not statistically different between the ArcRisk cohorts. In comparison with other European studies, the Mediterranean populations exhibit higher median concentrations of hexachlorobenzene (HCB), β-HCH, and dichlorodiphenyltrichloroethane with its metabolites (21). In the Mediterranean cohorts, several adverse health effects, like low birth weight, low birth head circumference, poor social behaviour, increased incidence of attention-deficit hyperactivity disorder, decreased cognitive skills, overweight and alterations of porphyrin, thyroid and liver metabolism have been related to exposure to organohalogens.

## Placental transporters

The important properties of placental transfer by passive diffusion are molecular weight, pK_a_, lipid solubility and protein binding. The placenta also expresses a large variety of transporter proteins, which modify placental transfer processes, and they may transfer foreign compounds such as therapeutic agents, environmental pollutants and chemical carcinogens bearing structural resemblance to their physiological substrates (22). Depending on the localization and function of transporter proteins, they may either increase or decrease foetal exposure to foreign compounds.

The distribution of contaminants between maternal blood, cord blood and placenta are usually related. If compounds are metabolized in the foetus or placenta, metabolites may accumulate and cause toxic effects, and foetal and maternal serum levels may differ (20). A strong correlation has been observed between concentrations in the maternal and foetal compartments for perfluorooctane sulfonate, perfluorooctanoic acid, PCB153, HCB, PCB180 and *p*,*p*′-dichlorodiphenyldichlorethylene (DDE) (23). Thus, even if the placenta cannot prevent the transfer of foreign chemicals into the foetal circulation, it can at least modify their transfer and toxicity.

The function of transporter proteins may cause person-to-person variation in foetal exposure to environmental contaminants. A significant number of protein carriers have been identified in the placenta, and it has been suggested that they may play a role in the uptake and/or efflux of MeHg complexes (24). The role of polymorphisms of ABC transporters as modifiers of prenatal exposure to MeHg has been studied (25) in two birth cohorts, one in Italy and Greece (PHIME) and the other in Spain (INMA). Polymorphisms (n=5) in the ABC genes ABCA1, ABCB1, ABCC1 and ABCC2 were analysed, and the findings showed the role of ABC transporters in MeHg accumulation. Cadmium may modulate foetal exposure to other harmful compounds transported by ABCG2, one of the main efflux transporters in human placenta, by inhibiting its activity (24). The metal salts methylmercury chloride (MeHgCl) and lead chloride (PbCl_2_) were not found to affect mRNA or protein expression of ABCG2, but cadmium chloride (CdCl_2_) inhibited its function. Further studies are needed to clarify whether this leads to elevated placental transfer of ABCG2 substrates (26).

## Toxicokinetic modelling of PCB153 and CoZMoMAN model

Systematic monitoring has been conducted for a short period compared to the entire contamination history, and it is possible to extrapolate body burden and exposure to the whole lifespan of the population under certain assumptions. The known disposition of PCB153 in the human body combined with population toxicokinetic modelling makes extrapolation possible to an acceptable accuracy (27). Birth during the 1960s and 1970s has led to high lifelong exposure, and body burdens remain elevated. Although lifelong exposure is lower for generations born after the 1960s, contamination during early childhood and possibly during the foetal period has been extremely high due to the high contamination levels of mothers leading to large exposure of the foetus during pregnancy and from contaminated milk during breastfeeding. Currently, the health risk of PCB153 in Arctic populations is estimated by using a toxicological cut-off point in lipids, that is, a benchmark dose level of 300 µg/kg plasma lipid, at which it is considered to pose no appreciable risk or minimal risk to human health (28).

Another model to understand the past and present human Polychlorinated biphenyl congener 153 (PBC) exposure is CoZMoMAN model (29), which was used in person-specific predictions of life course concentrations of PCBs in individual Norwegians by using dietary and lifestyle variables (30). The CoZMoMAN model was evaluated by reproducing measured time-variant concentrations of PCBs in environmental compartments, local food items and human breast milk. The rank correlation between measurements and predictions from both the CoZMoMAN model and regression analyses was strong (Spearman's r >0.67). Contamination histories for individuals predicted by the CoZMoMAN model revealed variation between study subjects, particularly in the timing of peak concentrations. The time-variant model CoZMoMAN has been useful in estimating prenatal, postnatal and childhood exposure to PCB153 under scenarios of hypothetical and realistic maternal fish consumption (31).

## Literature reviews

Reviews and meta-analyses of original scientific articles are needed to evaluate the potential health effects and their magnitude, and literature reviews were done in the ArcRisk project aimed to establish whether there are correlations between exposure to contaminants and detected health outcomes (32–35). Combining the published data was challenging, because of the use of different measurement scales of explanatory variables and the lack of necessary information in the study reports. Reporting on model results requires ancillary information such as tables that describe the basic data. Complexity and diversity across studies with regard to the selection of variables and reporting practices has made it difficult to combine and compare original studies. More on the reporting of descriptive statistics is needed. Among other things, the distributions of response and explanatory variables are needed when summarizing and meta-analysing the magnitude of effects.

A new method, the synthesis of regression coefficients, was developed to combine the findings across different published studies with statistical contents. This method helps in identifying significant findings from combined cohorts with identical variables (33). In ArcRisk, critical reviews were prepared based on original articles describing studies on PCBs (as a total sum of PCBs, a sum of more than six congeners, or PCB153) and DDT and related compounds (DDTs) in relation to weight and sex ratio at birth. These critical reviews (32–34) showed the following: a weak correlation between birth weight and exposure to PCBs, no correlation between birth weight and exposure to DDTs, and no correlation between the sex ratio of newborns and exposure to PCBs. The results are supported by data collected from 27 circumpolar jurisdictions of the eight Arctic countries, which showed that the contaminants present do not disrupt endocrine systems to the extent that sex ratios are affected (36,37).

The observation of Taylor et al. (38) confirmed this view in their study of POPs (organochlorine, organofluorine and organobromine compounds) and health outcomes related to type 1 and type 2 diabetes, and childhood obesity with type 2 diabetes. Only 43 studies were eligible in their meta-analysis out of 2,752 publications. Same difficulties were found in another study of association between environmental contaminants and health effects in indigenous populations in the Arctic (39). Difficulties in drawing conclusions included the small number of studies, studies restricted to a small number of regions and mixed results. They recommended further studies on the association between environmental contaminants and health with a wider geographical coverage. This was done within the framework of the European Union projects ENRIECO and OBELIX, and the effects of PCBs and DDE on birth weight were studied (32). This covered maternal and cord blood and breast milk samples in 15 study populations from 1990 through 2008. The meta-analysis including all cohorts indicated a birth weight decline of 150 g per 1 µg/l increase in PCB153, and DDE was associated with a 7-g decrease in birth weight. The findings suggest that low-level exposure to PCB impairs foetal growth, but that exposure to DDE does not.

## Toxicokinetic modelling and future risk prediction

Risk assessment of environmental pollutants requires data from different sources and methodology, for example, from in vivo toxicology, in vitro toxicology, mathematical modelling and quantitative methods, risk characterization of chemicals in food and diet, epidemiology, and the use of toxicogenomics. All these may form part of the multifaceted framework of evidence-based toxicology leading to a well-documented overall risk assessment process (1,40). The main challenge in traditional risk assessment is how to link external and internal doses. A modified approach based on the traditional risk assessment process has been introduced for quantitative risk estimates (27). This comprises three stages: extrapolation of exposure by pharmacokinetic modelling, incorporation of the reference dose and cancer slope factor, and estimation of hazard quotient (HQ) and life-time cancer risk. The only deviation from the traditional exposure assessment procedure is that the average daily and average life-time doses are calculated based on the extrapolation of contaminant concentrations in blood by toxicokinetic modelling. In this model, the total dose is a sum of all exposure pathways: inhalation, ingestion or dermal absorption, and these are all reflected in the total blood concentration of a chemical. Metabolism, excretion and accumulation in tissues other than blood complicate the issue.

**Fig. 1:**
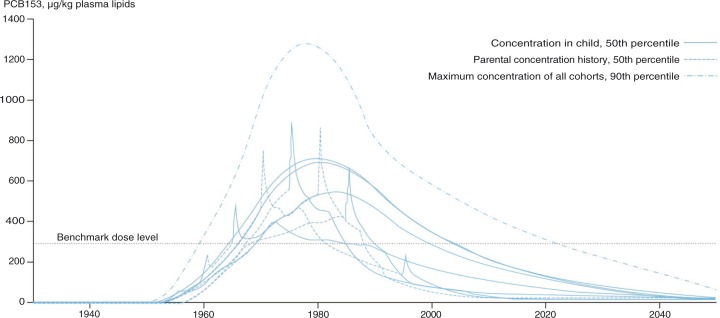
Extrapolated concentrations of PCB153 in pooled plasma lipids among pregnant Inuit women living in Nunavik (Quebec, Canada), Disko Bay (Greenland) and Nuuk (Greenland). Ref. (27), reprinted with permission from Elsevier.

Blood concentrations of polychlorinated biphenyl congener 153 (PCB153) were used to extrapolate body burden and exposure through the whole lifespan of the population using the one-compartment toxicokinetic model (Fig. 1) (27). By using risk characterization modelling, hazard quotient and cancer risk were estimated. The Abass et al.'s (27) study relied on the dose–response values (reference dose and cancer slope factor) established by the United State Environmental Protection Agency – Integrated Risk Information System. Non-cancer hazard and cancer risk estimation are widely accepted and commonly used in the field of chemical risk assessment. The next step should include the incorporation of dietary information as well as potential residential and personal exposure trends. Studies should also examine cumulative risk assessment for PCBs and other contaminants measured in human blood. The answers to the important questions – what is the total contaminant burden people acquire over their lifespan and what are the long-term health effects – require more multidisciplinary research, and the toxicokinetic modelling approaches presented above for PCB153 could be one means for estimating human health risk.

**Fig. 2:**
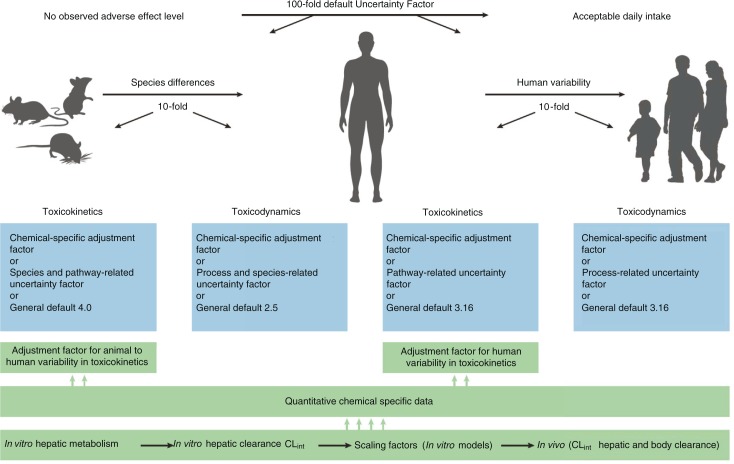
The subdivision of the 100-fold default uncertainty factor and the integration of in vitro data into human health risk assessment. Ref. (45), reprinted with permission from Elsevier.

## Toxicokinetic modelling and total risk estimation

Within the ArcRisk project, Čupr et al. (41) presented an approach to estimate the total risk of POPs. Bioaccumulation of the lipid-soluble POPs leads to high levels in breast milk; therefore, a new method was developed for the risk assessment of POPs for breastfeeding women. The method depends on the backward model for lifelong POPs exposure of breastfeeding women. The total human health risk of selected POPs in the form of a hazard index (HI) was calculated as the sum of individual HQs for selected POPs (according to the availability of reference dose values). The total risk HI shows that the main risk-posing group continues to be the PCBs (42). This new method presented for human health risk assessment of breastfeeding women is a useful tool when data from long-term biomonitoring epidemiologic studies are available. In addition, biomonitoring of breast milk is useful in evaluating internal exposure of humans to different chemical substances; and estimated total intake can be compared with changes in “food baskets” to predict the prevailing sources of exposure from different food types.

## The incorporation of in vitro mechanistic studies in human health risk assessment

The aim of in vitro characterization is to produce relevant information on metabolism and interactions to anticipate and ultimately predict what could happen in vivo in humans. To understand some of the factors related to environmental contaminant metabolism, there are several important points to consider, such as the metabolic stability of the compound, reactive metabolites, variation between mammalian species, human cytochrome P450 enzymes (CYPs, activation or detoxification), variation between individuals, subpopulations at increased risk and the overall process of human risk assessment. Examples of the incorporation of in vitro biotransformation studies into human health risk assessment were published by Abass et al. (43–45). In vitro metabolism of the pesticide benfuracarb was studied in liver microsomes from seven mammalian species to develop quantitative species-specific profiles and make risk assessment by interspecies comparisons. Analysis showed that benfuracarb was extensively metabolized with roughly similar profiles in different mammalian species in vitro, and there are quantitative interspecies differences in the metabolic profiles and kinetics of benfuracarb biotransformation.

Human responses to the toxicological effects of chemicals are often complicated by a substantial interindividual variability in toxicokinetics, of which metabolism is often the most important factor. Human variation and the contributions of human-CYP isoforms to in vitro metabolism of benfuracarb were, therefore, investigated (44), and kinetic parameters [K_m_, V_max_ and intrinsic clearance (CL_int_)] for carbofuran production in 10 hepatic samples varied 7.3-, 3.4- and 5.4-fold, respectively. Human CYP3A4 is the major enzyme and also the primary source of interindividual differences. For risk assessment, the quantitative in vitro chemical-specific data can then be scaled to determine the in vivo hepatic clearance (Fig. 2). This approach is used to extrapolate in vitro metabolic data to the in vivo situation and to translate interspecies and interindividual in vivo hepatic clearances into risk assessment of chemicals.

## Conclusions

According to the results of the ArcRisk project, current and future trends in contaminant levels may vary in different geographical areas and populations of the Arctic and Europe (new hot spots may develop). Extreme weather events associated with global climate change might also affect food and water security, possibly increasing the incidence of contaminated food items. It is also huge demand for the developing new approaches for the estimation of the magnitude of health effects of exposed populations, estimation of the effects of mixtures and also to use new-type methodologies for human health risk assessment. During the ArcRisk project, already some new approaches were developed.
